# Impact of pre-harvest fungicide application on the storage performance of tomato fruits

**DOI:** 10.1371/journal.pone.0308670

**Published:** 2024-08-08

**Authors:** Zhifu Lan, Jing Huang, Komivi S. Akutse, Yongwen Lin

**Affiliations:** 1 Zhangzhou Institute of Technology, Zhangzhou, China; 2 International Centre of Insect Physiology and Ecology (*icipe*), Nairobi, Kenya; 3 Unit for Environmental Sciences and Management, North-West University, Potchefstroom, South Africa; CPRI: Central Potato Research Institute, INDIA

## Abstract

To examine the impact of pre-harvest fungicide applications on the postharvest storage performance of tomato fruits, we measured the lycopene content, hardness, soluble solids content, rotting rate, and weight loss rate of the fruits, as well as conducted a sensory assessment. Protective and systematic fungicides were sprayed on tomatoes 20 days before harvest in order to prevent rotting and weight loss during storage. Our findings showed that, the fungicide-treated tomatoes had a significantly lower rotting rate of 16.00% and a weight loss rate of 3.96%. However the control group experienced 65.33% rotting rate and 6.90% weight loss rate on 12^th^ days of storage. Out of the pre-harvest applications, ‘Zineb’ a protective fungicide significantly delayed the loss of hardness and soluble solids accumulation in tomato fruits during storage, but it had no significant effect on lycopene content. On the other hand, when comparing to the examined treatment, the systemic fungicides did not have any significant effect on the postharvest storage performance of tomato fruits. Sensory evaluation results indicated that systemic fungicides improved the aroma of the fruits, while protective fungicides had a greater impact on the appearance and juiciness of the fruits. This study offers a potential novel solution for preserving fruits and vegetables which have been frequently infected by phytopathogens during storage, and consequently mitigate/reduce postharvest losses.

## Introduction

Fungicides play a crucial role in agricultural production [1–4]. They are primarily used to manage plant diseases by inhibiting the growth and reproduction of the causal pathogens, aiding crops in resisting against pathogens, and enhancing yield and quality [5–7]. Fungicides can also boost plant resistance, enabling them to grow normally in unfavorable environmental conditions [8]. Furthermore, fungicides can effectively manage the spread of diseases and minimize crop losses, thereby ensuring the stability and sustainable development of agricultural production [9]. Fungicides are categorized into systemic fungicides and protective fungicides [10, 11]. Systemic fungicides can be absorbed by plants and then transmitted to the lesions to eliminate pathogens. Common systemic fungicides include propiconazole, methyl thiophanate, and thiophanate-methyl. Protective fungicides form a film on the surface of plants to prevent fungal spores from invading [12–14]. These fungicides are highly effective in preventing the occurrence of disease [13, 14]. Commonly known protective fungicides include zinc dimethyldithiocarbamate, Bordeaux mixture, and chlorothalonil.

In addition to controlling plant diseases, fungicides can also regulate plant metabolism, which may impact the preservation of fruits [15–17]. For example, triazole fungicides, in addition to their fungicidal function, can also affect the metabolism of cytokinins in plants [18]. They inhibit the biosynthesis of gibberellins and auxins, while promoting the synthesis of abscisic acid and cytokinins [19]. However, improper or excessive use of triazole fungicides may inhibit crop growth and lead to issues such as fruit cracking, fruit drop, and deformed fruits [20]. Therefore, we hypothesized that fungicides may continually impact plants during the mid-to-late stages of their phenological growth. These fungicides could then further inhibit the development of postharvest diseases and consequently have a positive effect on the preservation or storage of postharvest fruits and vegetables.

To verify the above defined hypothesis, this study focused on tomato fruits, and assessed their key characteristics such as rotting rate, weight loss rate, hardness, soluble solids content, and lycopene content. The study aimed to comprehensively assess the impact of different fungicides applied before harvest on the quality of postharvest stored tomatoes. The preservation mechanism of residual fungicides, along with conducting sensory evaluations was also investigated. The main objective of this research was therefore to provide new insights into tomato postharvest preservation and theoretical/knowledge support for the development of scientific fungicide usage regimes.

## Materials and methods

### Pre-harvest fungicide treatment for tomatoes storage

Three types of systemic fungicides and three types of protective fungicides were selected for this study (Table 1). The fungicides were obtained from Sinochem Agro LTD., and their active ingredient and manufacturers are also summarized in Table 1. The fungicides were diluted with distilled water according to the multiples dilution/application rates of a specific fungicide as shown in Table 1. The tomato (*Solanum lycopersicum*) variety used in the experiment was “Hongfen #3”, obtained from the Vegetable Research Laboratory in the College of Horticulture, Fujian Agriculture and Forestry University. The tomato plants were grown in the greenhouse at 25 ± 2°C, relative humidity of 60 ± 5% and photoperiod of 12L: 12D in Zhangzhou, China. The prepared or diluted fungicide solutions were sprayed directly onto the surface of tomato fruits 20 days before harvesting. The fruit surface was sprayed until the liquid drips from it. The control treatments were sprayed with only sterile distilled water at the same rate. After harvesting, the fruits were stored separately as per the various treatments at room temperature (20 ± 5°C) with 25% relative humidity in a dark environment. The harvest (the collected tomato samples) was done at 1–2 days post-ripening of the fruits. The experiment was repeated five times at five different farms in randomized complete block design.

**Table 1:** Fungicides used in the experiment.

Fungicide type	Effective ingredient and concentration	Abbreviation	Formulation type	Application rate (dilution ratio)	Manufacturer
Systemic fungicides	70% Methyl Thiophanate	MT	Water dispersible	800	Nippon-Soda
	20% Propiconazole	PZ	Emulsion concentrate	1500	Syngenta
	2% Kasugamycin	FM	Aqueous solution	500	Hokko Chemical
Protective fungicides	80% Bordeaux Mixture	BM	Wettable powder	800	UPL
	80% Zineb	DN	Wettable powder	800	Bayer
	40% Carbendazim	CD	Suspension concentrate	800	Syngenta

### Measurement of postharvest characteristics of the stored tomato fruits

#### Rotting rate

Twenty healthy, pest-free tomatoes of similar size were selected from different treatment groups and placed in a storage room at 20 ± 5 °C with relative humidity not exceeding 30%, in darkness, for 15 days. The number of rotten tomatoes was counted every three days, and the rotten tomatoes were removed daily to prevent re-infection until the end of the 15 days. The experiment was repeated five times under a randomized complete block design.

In addition, to assess the effect of low temperature, 20 healthy, pest-free tomatoes of similar size were also selected from the different treatment groups and placed in a storage room at 20 ± 5 °C with a relative humidity of 30%, in darkness, for 12 days. Total tomatoes in each group were weighed prior to the storage and every three days until the 12 experimental days, and the mean value was calculated from the recorded data. At 2-day intervals, five tomato samples were randomly selected from each experimental group. Two positions were selected at the equator of each fruit as measurement points, and the firmness of each fruit was measured three times using a texture analyzer (ST-16A, Shandong, China) where prop size was used. The average value was then calculated to represent the fruit hardness. The experiment was repeated five times.

#### Content of soluble solids and lycopene of the stored tomatoes

Three tomatoes were randomly selected from each treatment group and homogenized at every two days until the end of the experiment. The soluble solids content of the juice was measured using a digital refractometer (DigiPol-R200, Shanghai, China). In addition, the three randomly selected tomatoes from each group for homogenization at 2-day intervals, were also weighed at approximately 0.80 g. After homogenization, 4 mL of extraction solution (n-hexane: acetone: ethanol, 2:1:1 by volume) was added to the tomato juice. After complete dissolution, the solution was left to stand in an ice bath for 10 minutes. The supernatant was then tested using n-hexane as a blank, and the absorbance was measured at a wavelength of 472 nm to determine the lycopene content using a spectrophotometer (SH-6600, Jiangsu, China).

#### Sensory evaluation

Ten adults’ panelists (5 males and 5 females) were chosen as sensory assessors. Each portion of tomato fruits was divided into five parts and given to each member of the panelists. The panelists rated each fruit based on the different sensory attributes: color, shape, aroma, taste, and juice, and then submitted the average value of the various assessed parameters. The scoring criteria for evaluating tomato sensory attributes were presented in Table 2.

**Table 2:** Sensory traits and scores for tomato.

Sensory traits	Score
Color	Bright to dull color, 9–1 point
Aroma intensity	Very strong to very week, 9–1 point
Taste	Very delicious to acor, 9–1 point
Mouth feeling	Very delicate to coarse, 9–1 point
Juice abundance	Juicy to juiceless

#### Statistical analysis

The significant differences in the mean values of the measured/assessed post-harvest characteristics or parameters were analyzed using an analysis of variance (ANOVA) with GraphPad Prism 9.0 software. The Tukey test was performed to separate the means whenever there were significant differences between treatments (P ≤ 0.05).

## Results

### Impact of pre-harvest fungicide application on tomato decay incidence during storage

**Fig 1:**
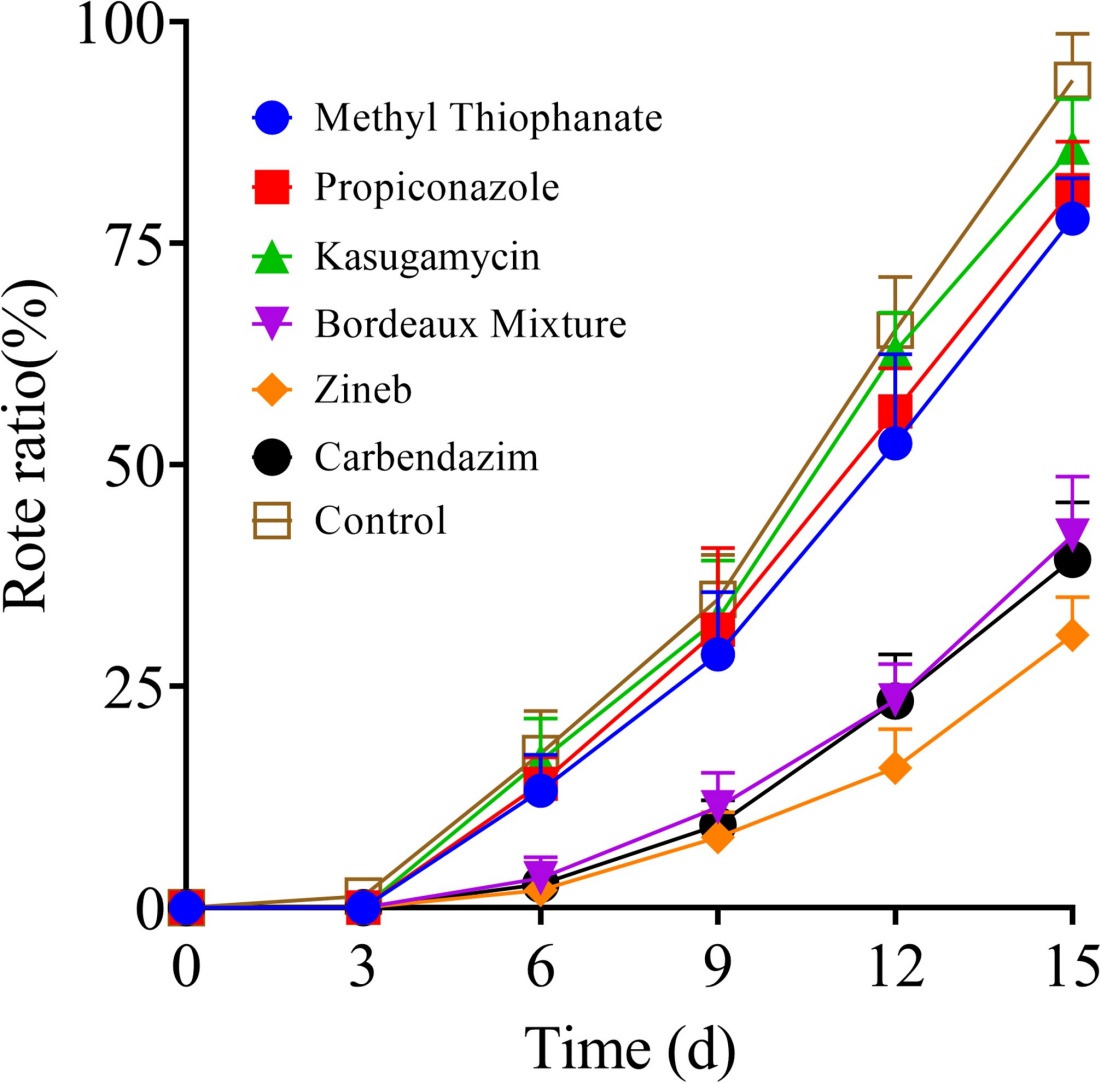
Impact of the various fungicides on the rot rate of tomato fruits during storage at room temperature (20 ± 5 °C) for 15 days post-treatment.

The results showed that the decay rate of tomato fruits increased during the storage period for all treatments (Fig 1). However, the rate of decay escalation was notably slower in tomato fruits treated with protective fungicides such as Zineb, Bordeaux mixture, and carbendazim, compared to the control and systemic fungicide treatments. A comprehensive comparison of the tomato fruits treated with systemic fungicides and those exposed to protective fungicides for 20 days before harvesting revealed significant differences starting from the 6^th^ day post-storage time. At day 6, the decay rates of the analyzed tomato fruits showed a gradient from low to high as follows: Zineb group (2.00%) < Bordeaux mixture group (3.33%) < carbendazim group (3.45%) < methyl thiophanate group (13.33%) < propiconazole group (14.00%) < kasugamycin group (16.67%) < control group (17.33%). On the 12^th^ and 15^th^ days post-storage, the decay rates in the Zineb group were 16.00% and 30.67%, respectively, which were significantly (*P < 0*.*0001*) lower than the rates obtained in the other groups during the same period (Fig 1). These findings showed that applying protective fungicides 20 days before harvest has a beneficial inhibitory effect on post-harvest tomato decay. Zineb showed superior long-term antibacterial effects compared to Bordeaux mixture and carbendazim, which were also found to be protective fungicides. Given that both the systemic fungicides and control groups experienced decay rates exceeding 75.00% within the first 15 days of storage, subsequent measurements were limited to the 12^th^ day.

### Impact of pre-harvest fungicide application on the rate of weight loss in stored tomato fruits

**Fig 2:**
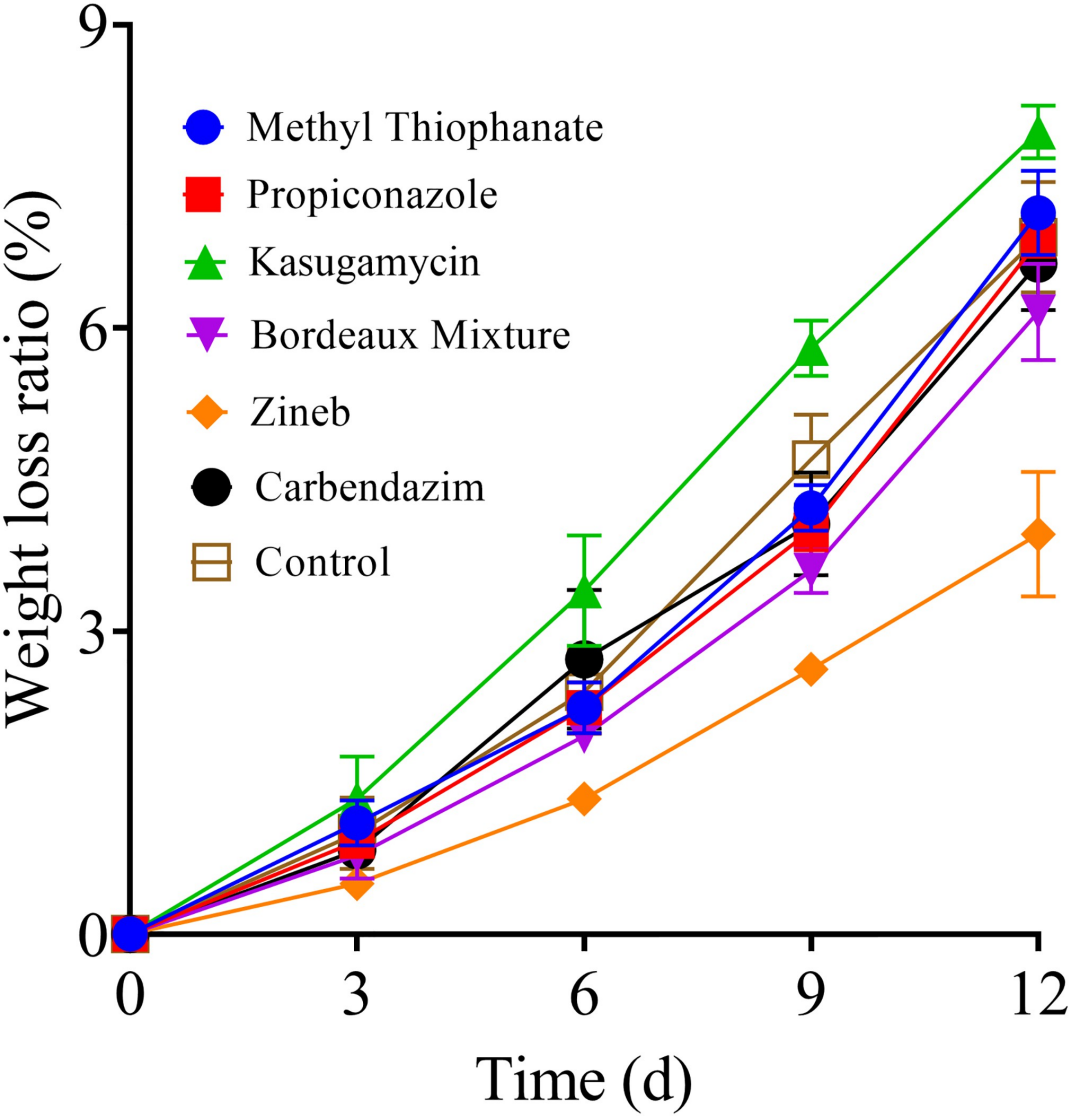
Impact of the various fungicides on the weight loss of tomato fruits during storage at room temperature (20 ± 5 °C) for 12 days post-treatment.

As represented in Fig 2, starting from day 6 post-storage, the rate of weight loss in the stored tomato fruits was significantly lower in the Zineb group as compared to the other treatment groups, while the weight loss rate in the kasugamycin group was significantly higher than the ones recorded in the other groups (*P < 0*.*0001*). On day 12, the weight loss rates of the tested tomatoes were raged from the lowest to the highest as follows: Zineb group (3.96%) < Bordeaux mixture group (6.16%) < carbendazim group (6.19%) < methyl thiophanate group (6.86%) < control group (6.90%) < propiconazole group (7.14%) < kasugamycin group (7.94%). The weight loss rate of the Zineb group was significantly lower than that of the other groups, while the kasugamycin and propiconazole groups showed significantly higher rates than the control group (Fig 2). These results indicate that the pre-harvest application of Zineb has a significant inhibitory effect on the weight loss of the stored tomatoes, while systemic fungicides such as kasugamycin and methyl tolylfluoroacetate could contribute to the tomato fruits weight loss during storage.

### Impact of pre-harvest fungicide application on the firmness of stored tomato fruits

**Fig 3:**
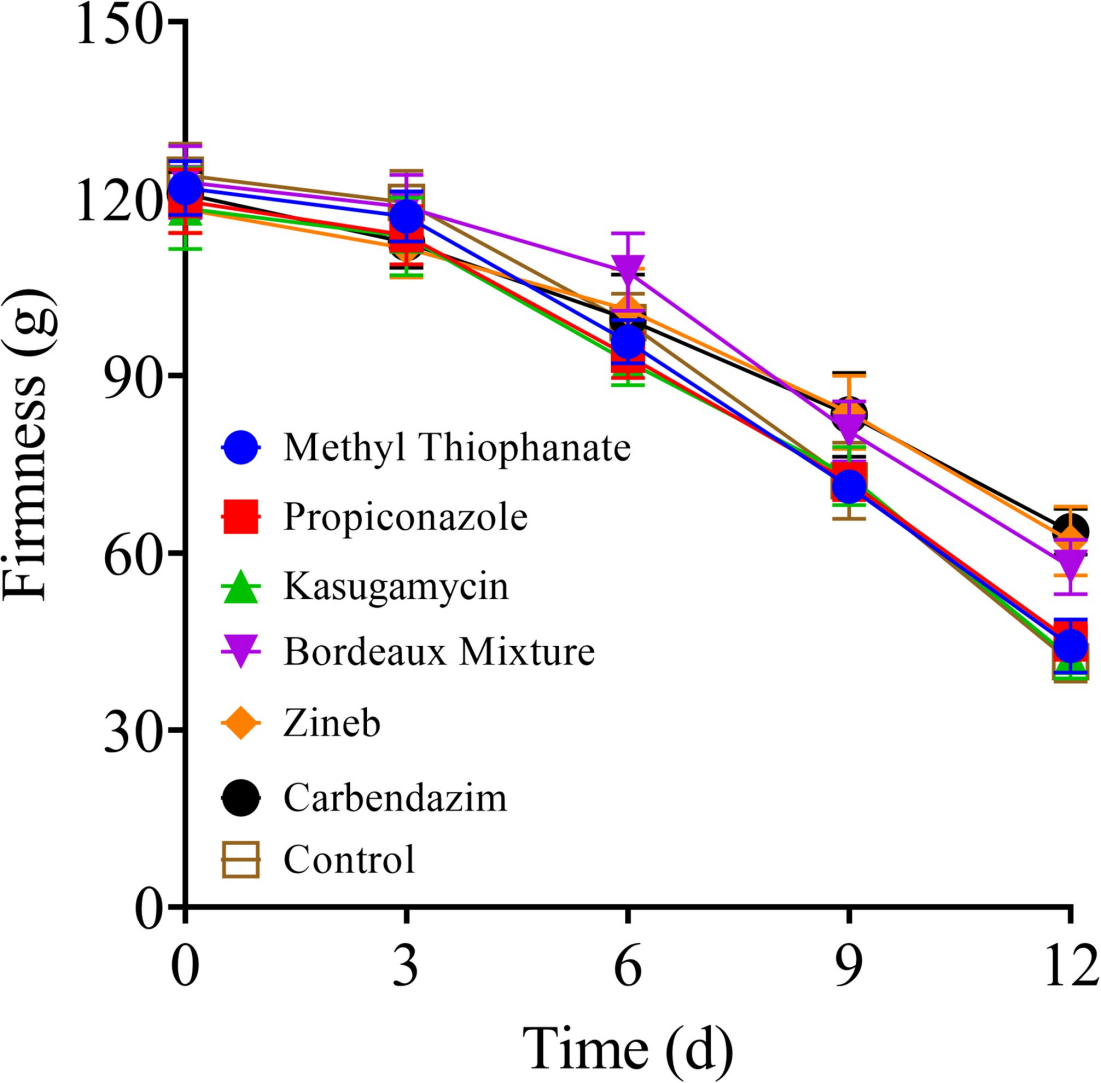
Effect of the various fungicides on the firmness of tomato fruits during storage at room temperature (20 ± 5 °C) for 12 days post-treatment.

As reported in Fig 3, starting from day 9 post-storage, the firmness of the tomato fruits treated with protective fungicides was significantly higher than that of the control and systemic fungicide groups (*P* < 0.05). Especially on day 12, the hardness of the stored tomato fruits in Zineb and Bordeaux mixture groups was 62.00 g and 57.60 g, respectively, which were significantly higher than that of the other groups (*P* < 0.0001). This indicated that the pre-harvest applications of protective fungicides significantly inhibited the softening of the stored tomato fruits.

### Impact of pre-harvest fungicide application on the soluble solids content of stored tomato fruits

**Fig 4:**
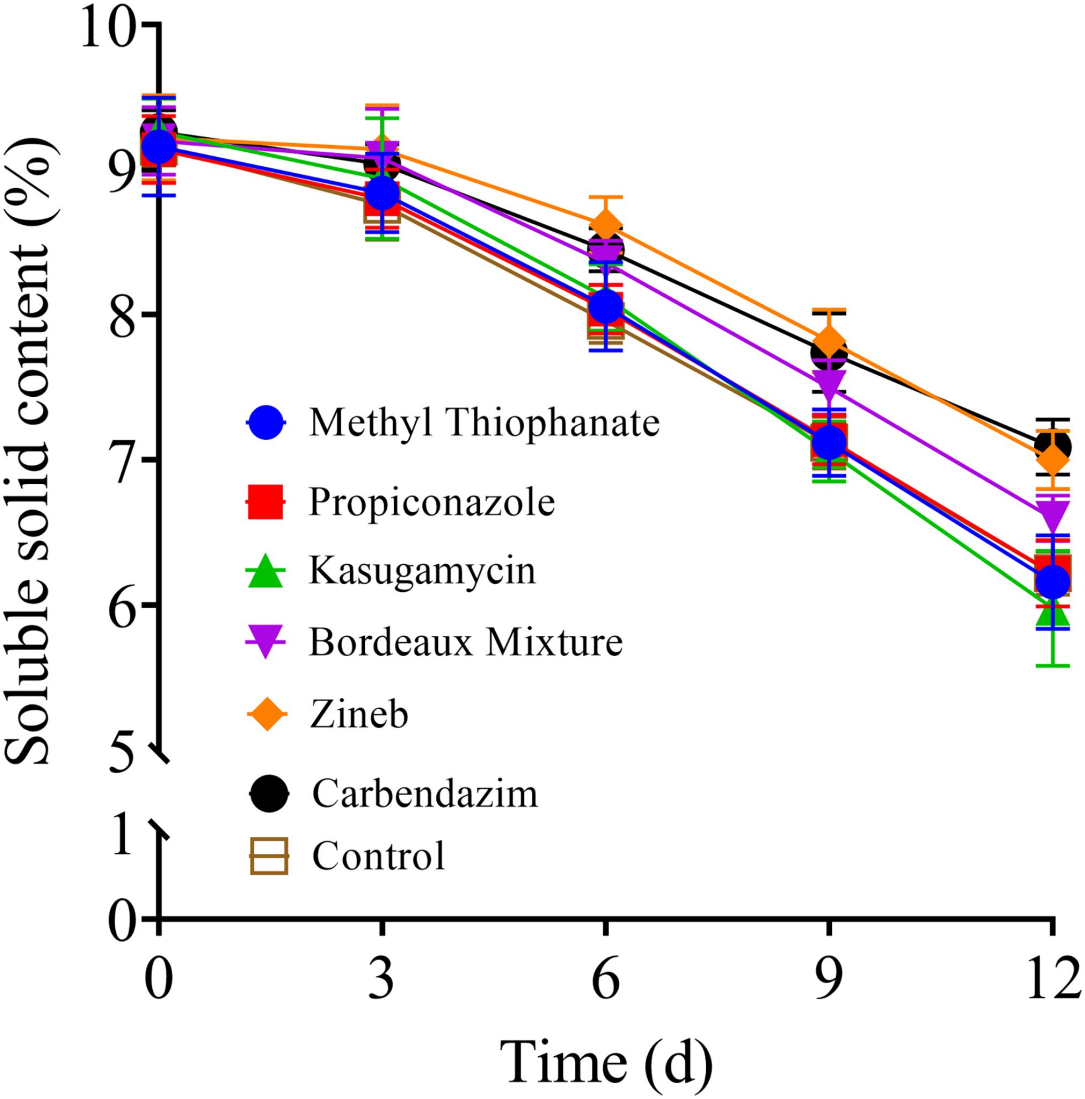
Effect of the various fungicides on the soluble solids content of tomato fruits during storage at room temperature (20 ± 5 °C) for 12 days post-treatment.

The results also showed that the soluble solids content of the stored tomatoes decreased over time in storage (Fig 4). However, the Zineb treatment group exhibited a slower rate of decline compared to the other treatment groups. The soluble solids content of the tomato fruits sprayed with Zineb on days 6, 9, and 12 of storage were 8.59%, 7.83%, and 7.01%, respectively, which were significantly (*P* < 0.05) higher than that of the fruits sprayed with systemic fungicides during the same storage period (Fig 4). These results indicate that Zineb has a significant inhibitory effect on the loss of soluble solids in the stored tomato fruits compared to the control and other fungicides.

### Impact of pre-harvest fungicide application on the lycopene content of stored tomato fruits

**Fig 5:**
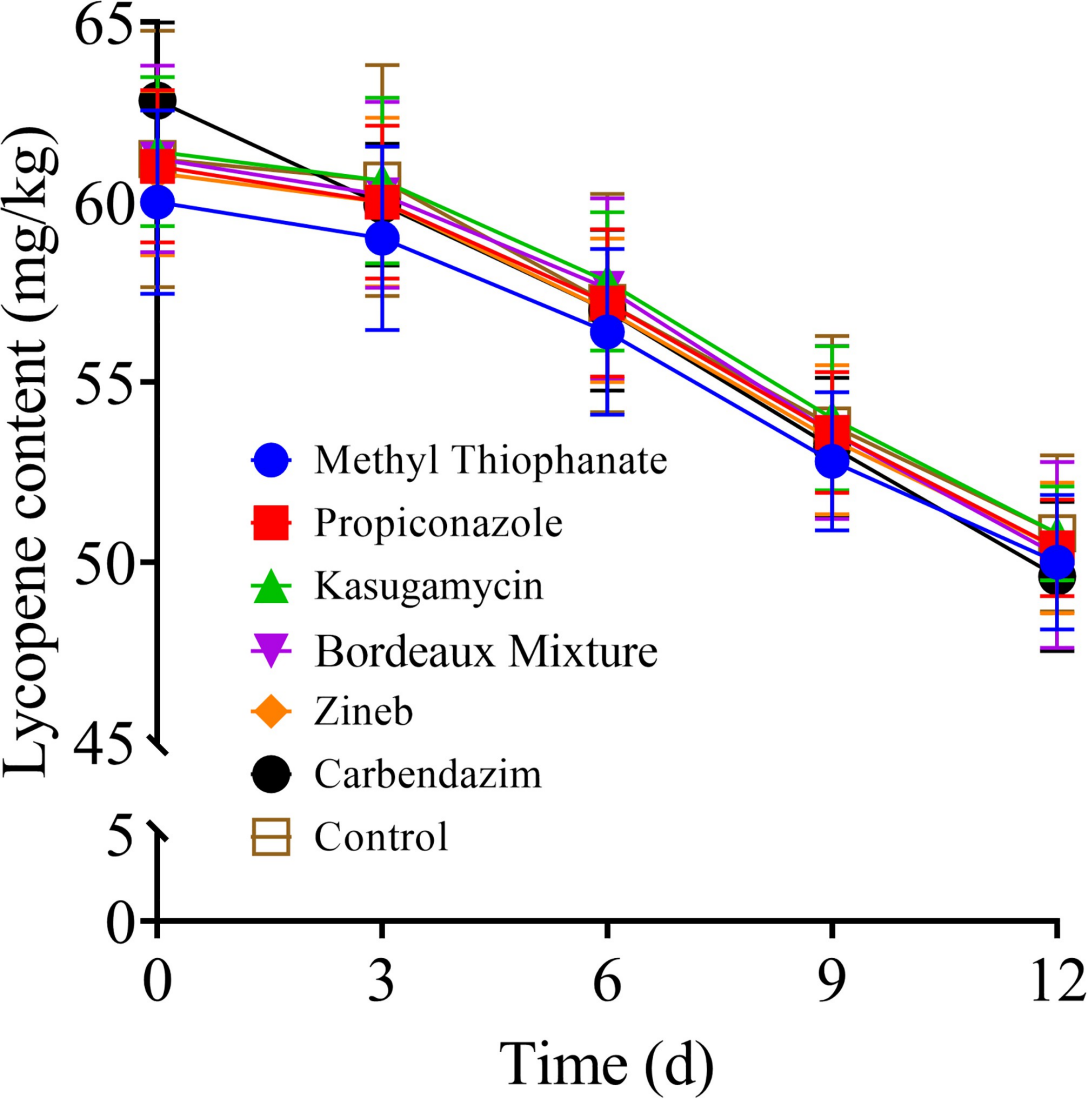
Effect of the various fungicides on the lycopene content of tomato fruits during storage at room temperature (20 ± 5 °C) for 12 days post-treatment.

As observed in Fig 5, the lycopene content of tomato fruits decreased over time in storage. The results indicated that there was no significant difference in the lycopene content of the stored tomato fruits among the various treatment groups at the different storage times (*P* > 0.05). This result suggests that the application of different types of fungicides 20 days before harvest did not have any impact on the lycopene content of the tomato fruits during storage.

### Sensory analysis

**Fig 6:**
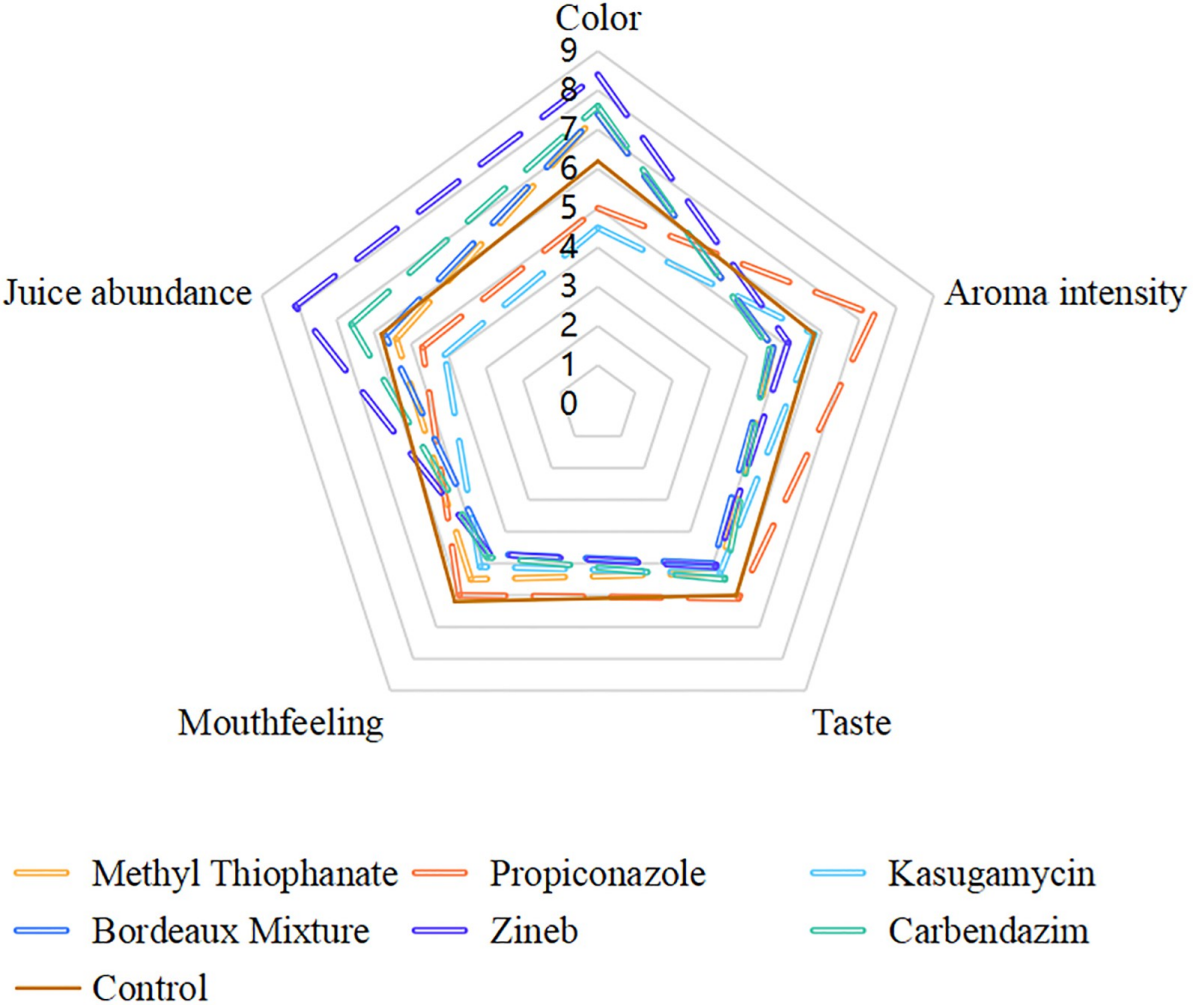
Sensory evaluation of tomatoes harvested immediately after application of the different fungicides using synthetical score method.

The results of the sensory analysis, as indicated in Fig 6, revealed distinct variations among the various treatment groups. However, the Zineb-treated tomato fruits were rated the highest for color, achieving a score of 8.4, surpassing the propiconazole and kasugamycin treatments, which scored 5 and 4.5, respectively, and outperformed the control group’s score of 6.2. Regarding juice content, the Zineb group also outperformed all the other treatment groups with a score of 8.1, notably higher than the propiconazole and kasugamycin groups, which scored 4.7 and 4.1, respectively, and exceeded the control’s score of 5.8. In the category of aroma intensity, the propiconazole group was rated superior, scoring 7.4, which was over 1.5 points higher as compared to the scores of the other groups. While the propiconazole group also had the highest ratings for taste and texture, the margin was not considerably different from the other treatments. These findings suggested that protectant fungicides such as Zineb have a greater influence on the visual attributes of the stored tomato fruits, whereas systemic fungicides like propiconazole affected more profoundly the aroma (Fig 6).

## Discussion

The use of pre-harvest and post-harvest fungicides is essential in agricultural products quality, with post-harvest application having the greatest impact on consumers. Studies have shown that most fungicides used for post-harvest product preservation have a certain level of chronic toxicity to human body/health, including fruit wax [21–23]. The combined impact of residual of pre-harvest fungicides and post-harvest fungicides has led to significant improvements in food safety [24]. This study investigated the impact of various pre-harvest fungicides on the storage quality of tomato fruits. The results indicated that the application of protective fungicides before harvest successfully prevented rotting and weight loss of the stored tomato fruits. Zineb exhibited superior antibacterial effects compared to Bordeaux mixture and carbendazim. These results are consistent with previous research findings, which demonstrated that the pre-harvest application of Zineb significantly reduced rotting of tomato fruits during storage [25]. Furthermore, the study also found that the tomatoes treated with Zineb had higher firmness, soluble solid content, and higher sensory analysis scores [26]. This may be attributed to the preserving effect of Zineb on the tomato fruit quality.

Compared to systemic fungicides, protective fungicides primarily create a protective film on the plant surface to prevent pathogen invasion [12]. The effectiveness of preservation achieved using protective fungicides before harvest may be attributed to the formation of a protective layer on the fruit surface. This layer might effectively prevent pathogen invasion through the skin, as most post-harvest diseases are caused by environmental pathogens [27]. Additionally, the protective fungicide layer could also prevent fruit moisture evaporation, which may explain the reduced weight loss and delayed softening of the stored fruits [28]. Systemic fungicides are absorbed by plants and transported within the plant, inhibiting or killing pathogens inside the plant. However, their effectiveness or protection efficacy against pathogens that have already entered the plant is limited [29]. This is consistent with previous research findings, such as the study conducted by Weitan *et al*. (2024), which demonstrated that systemic fungicides had limited effects in inhibiting pathogens, while protective fungicides effectively reduced rotting rates [30].

Furthermore, in addition to its fungicidal effect, the protective fungicide Zineb also provide plants with essential zinc elements [31, 32]. Zinc is an essential trace element for plants, playing a key role in the synthesis of various enzymes and the regulation of gene expression [33–35]. The zinc ions in Zineb can bind to the cell walls of pathogens, disrupting their structure and inhibiting their growth as observed in our study. This may be therefore one of the key factors contributing to Zineb’s ability to preserve the quality of tomato fruits during storage.

The results of this study indicate that the pre-harvest use of protective fungicides, particularly Zineb, could significantly improve the post-harvest storage time and preservation of the fruits quality. These findings imply that applying fungicides using the appropriate techniques or at the right time could be an effective strategy to minimize their application frequencies. Consequently, this research contributes to potential novel perspectives and approaches to quality improvement/preservation of tomatoes.

However, it is important to recognize that the experimental outcomes were obtained under controlled conditions, which might affect the efficacy of the approach or introduce certain constraints to their applicability under different field conditions [36]. Therefore, future studies should aim to replicate these experiments across different geographical or agroecological locations, using a range of tomato varieties, and under varying climatic conditions to establish the robustness, effective application and wider relevance of these findings. Additionally, there is a scope to delve deeper into the specific effects Zineb has on tomato fruit quality, nutritional values, and to evaluate the potential synergistic benefits of using Zineb in combination with other fungicides.

## References

[1] Hazra R, Roy J, Jiang L, Webster D, Rahman M, Quadir M. Biobased, macro-, and nanoscale fungicide delivery approaches for plant fungi control. ACS Appl Bio Mater. 2023;17:2698–711. doi: 10.1021/acsabm.3c00171 37405899

[2] Burandt Q, Deising H, von Tiedemann A. Further limitations of synthetic fungicide use and expansion of organic agriculture in Europe will increase the environmental and health risks of chemical crop protection caused by copper-containing fungicides. Environ Toxicol Chem. 2024;43(1):19–30. doi: 10.1002/etc.5766 37850744

[3] Jørgensen L, van den Bosch F, Oliver R, Heick T, Paveley N. Targeting fungicide inputs according to need. Annu Rev Phytopathol. 2017;55:181–203. doi: 10.1146/annurev-phyto-080516-035357 28525306

[4] Quesada-Ocampo L, Parada-Rojas C, Hansen Z, Vogel G, Smart C, Hausbeck M. Phytophthora capsici: Recent progress on fundamental biology and disease management 100 years after its description. Annu Rev Phytopathol. 2023;61:185–208. doi: 10.1146/annurev-phyto-021622-103801 37257056

[5] Ojiambo P, Paul P, Holmes G. A quantitative review of fungicide efficacy for managing downy mildew in cucurbits. Phytopathology. 2010;100:1066–76. doi: 10.1094/PHYTO-12-09-0348 20839942

[6] Dowling M, Peres N, Villani S, Schnabel G. Managing colletotrichum on fruit crops: A "Complex" challenge. Plant Dis. 2020;104(9):2301–16 doi: 10.1094/PDIS-11-19-2378-FE 32689886

[7] Beckerman J, Palmer C, Tedford E, Ypema H. Fifty years of fungicide development, deployment, and future use. Phytopathology. 2023;113(4):694–706. doi: 10.1094/PHYTO-10-22-0399-IA 37137816

[8] Khoshru B, Mitra D, Joshi K, Adhikari P, Rion M, Fadiji A. Decrypting the multi-functional biological activators and inducers of defense responses against biotic stresses in plants. Heliyon. 2023;9(3):e13825. doi: 10.1016/j.heliyon.2023.e13825 36873502 PMC9981932

[9] Xylia P, Chrysargyris A, Shahwar D, Ahmed ZFR, Tzortzakis N. Application of rosemary and Eucalyptus essential oils on the preservation of cucumber fruit. Horticulturae. 2022;8(9):774.

[10] Ayesha M, Suryanarayanan T, Nataraja K, Prasad S, Shaanker R. Seed treatment with systemic fungicides: Time for review. Front Plant Sci. 2021;12:654512. doi: 10.3389/fpls.2021.654512 34408757 PMC8365024

[11] Romanazzi G, Feliziani E, Sivakumar D. Chitosan, a biopolymer with triple action on postharvest decay of fruit and vegetables: Eliciting, antimicrobial and film-forming properties. Front Microbiol. 2018;9:2745. doi: 10.3389/fmicb.2018.02745 30564200 PMC6288236

[12] Yamada S. Protective fungicide applications for citrus disease. Japan Agricultural Research Quarterly. 1973;7(2):98–104.

[13] El-Baky N, Amara A. Recent approaches towards control of fungal diseases in plants: An updated review. J Fungi (Basel). 2021;7(11):900. doi: 10.3390/jof7110900 34829188 PMC8621679

[14] Deresa E, Diriba T. Phytochemicals as alternative fungicides for controlling plant diseases: A comprehensive review of their efficacy, commercial representatives, advantages, challenges for adoption, and possible solutions. Heliyon. 2023;9(3):e13810. doi: 10.1016/j.heliyon.2023.e13810 36879959 PMC9984788

[15] Elhamouly N, Hewedy O, Zaitoon A, Miraples A, Elshorbagy O, Hussien S, et al. The hidden power of secondary metabolites in plant-fungi interactions and sustainable phytoremediation. Front Plant Sci. 2022;13:1044896. doi: 10.3389/fpls.2022.1044896 36578344 PMC9790997

[16] Baskaran P, Jayabalan N. Effect of growth regulators on rapid micropropagation and psoralen production in Psoralea corylifolia L. Acta Physiologiae Plantarum. 2008;30(3):345–55.

[17] Junqueira V, Müller C, Rodrigues A, Amaral T, Batista P, Silva A, et al. Do fungicides affect the physiology, reproductive development and productivity of healthy soybean plants? Pesticide Biochemistry and Physiology. 2021;172:104754. doi: 10.1016/j.pestbp.2020.104754 33518047

[18] Matin M, Matin P, Rahman M, Ben Hadda T, Almalki F, Mahmud S. Triazoles and their derivatives: chemistry, synthesis, and therapeutic applications. Front Mol Biosci. 2022;9:864286. doi: 10.3389/fmolb.2022.864286 35547394 PMC9081720

[19] Berry P, Spink J. Understanding the effect of a triazole with anti-gibberellin activity on the growth and yield of oilseed rape (*Brassica napus*). J Agri Sci. 2009;147(3):273–85.

[20] Gomathinayagam M, Jaleel C, Lakshmanan G, Panneerselvam R. Changes in carbohydrate metabolism by triazole growth regulators in cassava (*Manihot esculenta* Crantz); effects on tuber production and quality. C R Biol. 2007;330(9):644–55. 17720581 10.1016/j.crvi.2007.06.002

[21] Kumar P, Ahlawat S, Chauhan R, Kumar A, Singh R, Kumar A. In vitro and field efficacy of fungicides against sheath blight of rice and post-harvest fungicide residue in soil, husk, and brown rice using gas chromatography-tandem mass spectrometry. Environ Monit Assess. 2018;190(9):503. doi: 10.1007/s10661-018-6897-7 30088099

[22] Donnarumma L, Pompi V, Rossi E, Carfì F. Fungicide residues in pears and apples after post harvest treatments by drencher. Commun Agric Appl Biol Sci. 2005;70(4):1053–8. 16628954

[23] Imura N, Ae M, Hoshino R, Abe M, Yamamuro T, Oyama K, et al. Membrane hyperpolarization and depolarization of rat thymocytes by azoxystrobin, a post harvest fungicide. Chem Biol Interact. 2019;300:35–9. doi: 10.1016/j.cbi.2019.01.006 30629953

[24] Xylia P, Chrysargyris A, Ahmed ZFR, Tzortzakis N. Application of rosemary and Eucalyptus essential oils and their main component on the preservation of apple and pear fruits. Horticulturae. 2021;7(11):479.

[25] Domínguez I, Ferreres F, Riquelme FPd. Influence of preharvest application of fungicides on the postharvest quality of tomato (*Solanum lycopersicum* L.). Postharvest Biology and Technology. 2012;72:1–10.

[26] Saeedi S, Shokrzadeh M. Effects of washing, peeling, storage, and fermentation on residue contents of carbaryl and mancozeb in cucumbers grown in greenhouses. Toxicol Ind Health. 2016;32(6):1135–42. doi: 10.1177/0748233714552295 25342670

[27] Kaur N, Shahwar D, Hassan FE, Ahmed ZFR. Antioxidant and antibacterial activities of date palm fruit (*Phoenix dactylifera* L.) in response to postharvest application with natural elicitors. Acta Horticulturae. 2023;1364:187–194.

[28] Abdullah AH, Awad-Allah MAA, Abd-Elkarim NAA, Ahmed ZFR, Taha EMA. Carboxymethyl Cellulose from Banana Rachis: A Potential Edible Coating to Extend the Shelf Life of Strawberry Fruit. Agriculture. 2023;13(5):1058.

[29] Hahn M. The rising threat of fungicide resistance in plant pathogenic fungi: *Botrytis* as a case study. J Chem Biol. 2014;7(4):133–41. 25320647 10.1007/s12154-014-0113-1 PMC4182335

[30] Weitang S, Ligang Z, Chengzong Y, Xiaodong C, Liqun Z, Xili L. Tomato *Fusarium wilt* and its chemical control strategies in a hydroponic system. Crop Protection. 2004;23(3):243–7.

[31] Luo Y, Yao A, Tan M, Li Z, Qing L, Yang S. Effects of manganese and zinc on the growth process of Phytophthora nicotianae and the possible inhibitory mechanisms. PeerJ. 2020;8:e8613. doi: 10.7717/peerj.8613 32117636 PMC7036275

[32] Calatayud Á, Barreno EF. Spraying with zineb increases fruit productivity and alleviates oxidative stress in two tomato cultivars. Photosynthetica. 2000;38:149–54.

[33] Luis G, Hernández C, Rubio C, González-Weller D, Gutiérrez Á, Revert C, et al. Trace elements and toxic metals in intensively produced tomatoes (*Lycopersicon esculentum*). Nutr Hosp. 2012;27(5):1605–9. 23478712 10.3305/nh.2012.27.5.5944

[34] Parra-Torrejón B, Cáceres A, Sánchez M, Sainz L, Guzmán M, Bermúdez-Perez F. Multifunctional nanomaterials for biofortification and protection of tomato plants. Environ Sci Technol. 2023;57(40):14950–60. doi: 10.1021/acs.est.3c02559 37753594 PMC10569043

[35] Alkaabi R, Matar HHB, Karthishwaran K, Ahmed ZFR, Kurup S, Alyafei MS, et al. Extraction of nutrients from Rumex vesicarius, a wild indigenous edible plant from United Arab Emirates. Notulae Scientia Biologicae. 2023;15(3):11658.

[36] Nadeem A, Ahmed ZFR, Hussain SB, Omar AE-DK, Amin M, Javed S. On-tree fruit bagging and cold storage maintain the postharvest quality of mango fruit. Horticulturae. 2022;8(9):814.

